# A2aR antagonists: Next generation checkpoint blockade for cancer immunotherapy

**DOI:** 10.1016/j.csbj.2015.03.008

**Published:** 2015-04-08

**Authors:** Robert D. Leone, Ying-Chun Lo, Jonathan D. Powell

**Affiliations:** Sidney Kimmel Comprehensive Cancer Research Center, Department of Oncology, Johns Hopkins University School of Medicine, Baltimore, MD 21287, USA

**Keywords:** A2aR, adenosine A2a receptor, APC, antigen presenting cell, CTLA-4, cytotoxic T-lymphocyte-associated protein 4, DLBCL, diffuse large B-cell lymphoma, Hif1-alpha, hypoxia inducible factor-1 alpha, LAG-3, lymphocyte-activation gene 3, NSCLC, non-small cell lung cancer, ORR, overall response rate, OS, overall survival, PD-1, programmed cell death 1, PD-L1, programmed cell death ligand 1, TFS, tumor free survival, TIM-3, T-cell immunoglobulin domain and mucin domain 3, T_reg_, regulatory T cell, A2a adenosine receptor, Immune checkpoint, Tumor, T cell, Immunotherapy, PD-1

## Abstract

The last several years have witnessed exciting progress in the development of immunotherapy for the treatment of cancer. This has been due in great part to the development of so-called checkpoint blockade. That is, antibodies that block inhibitory receptors such as CTLA-4 and PD-1 and thus unleash antigen-specific immune responses against tumors. It is clear that tumors evade the immune response by usurping pathways that play a role in negatively regulating normal immune responses. In this regard, adenosine in the immune microenvironment leading to the activation of the A2a receptor has been shown to represent one such negative feedback loop. Indeed, the tumor microenvironment has relatively high concentrations of adenosine. To this end, blocking A2a receptor activation has the potential to markedly enhance anti-tumor immunity in mouse models. This review will present data demonstrating the ability of A2a receptor blockade to enhance tumor vaccines, checkpoint blockade and adoptive T cell therapy. Also, as several recent studies have demonstrated that under certain conditions A2a receptor blockade can enhance tumor progression, we will also explore the complexities of adenosine signaling in the immune response. Despite important nuances to the A2a receptor pathway that require further elucidation, studies to date strongly support the development of A2a receptor antagonists (some of which have already been tested in phase III clinical trials for Parkinson Disease) as novel modalities in the immunotherapy armamentarium.

## 1 Introduction

The immune system has evolved an array of regulatory mechanisms to protect against tissue damage from autoimmunity or during active response to pathogen. Both central mechanisms (negative selection in the thymus) and peripheral mechanisms (e.g., deletion, anergy, and regulatory T cells (T_regs_)) contribute to establishing self-tolerance. Nonetheless, inherent in active immune responses against pathogens are inhibitory and negative feedback pathways which prevent collateral damage. Included in these protective mechanisms are a broad array of inhibitory receptors that are upregulated on lymphocytes during an active immune response. These inhibitory receptors and their related signaling networks, known as “immune checkpoint pathways,” provide a negative feedback mechanism that is crucial for immunoregulation and protection of tissues from an overexuberant inflammatory response.

While the negative feedback loops created by checkpoint pathways are critical in modulating excessive inflammation, they are also subject to dysregulation in the presence of cancer and provide tumors with a means of immune evasion. Recently, clinical trials have confirmed that blockade of immune checkpoint pathways mediated by the CTLA-4 and PD-1 receptors can unleash an endogenous immune attack, leading to significant responses and long-term remissions in multiple solid tumor types [1–3]. In fact, antibody-mediated blockade of CTLA-4 and PD-1, alone or in combination, have led to unprecedented responses in refractory, metastatic melanoma, as well as in renal cell carcinoma and non-small cell lung cancer. The success of checkpoint blockade in these trials has been a major step forward in the development of immunotherapy for the treatment of cancer, confirming the clinical importance of tumor immune evasion through usurping fundamental pathways of immune regulation. With the success of CTLA-4 and PD-1 inhibition in clinical trials, significant effort has focused on uncovering other targetable checkpoint pathways active in the tumor microenvironment. In this regard, adenosine signaling through the A2a receptor has been found to function as one such promising negative feedback loop [4–7]. As we shall discuss, while the effects of A2a receptor inhibition in antitumor therapy can behave as a double-edged sword (depending on the degree and, likely, the duration of signaling blockade), preclinical studies have confirmed that blockade of A2a receptor activation has the ability to markedly enhance anti-tumor immunity. As such, A2a receptor blockade represents the potential next generation of immune checkpoint inhibition in cancer immunotherapy.

## 2 CTLA-4 and PD-1 and the arrival of cancer immunotherapy

Immune checkpoint pathways such as those mediated by CTLA-4 and PD-1 receptors are critical aspects of normal physiologic function. The hallmark of these pathways is the generation of a negative feedback loop that preserves self-tolerance and prevents excessive tissue damage in the setting of immune response. The pathways regulated by CTLA-4 and PD-1 receptors have somewhat distinct modes of action on the immune response [5,8]. CTLA-4 is upregulated during initial activation of effector T cells and is thought to counteract the activity of the co-stimulatory receptor CD28 by two mechanisms. By out-competing the lower affinity CD28 for engagement of shared, cognate ligands B7.1 and B7.2 on antigen presenting cells (APCs), as well as by providing a direct inhibitory signal, the CTLA-4 receptor dampens the effector T cell activation sequence [5,9–11]. CTLA-4 is also strongly expressed on regulatory T cells and enhances immunosuppression through enhancing T_reg_ activity and proliferation [12]. Like CTLA-4, PD-1 is induced upon effector T cell activation and is also highly expressed on T_regs_ [13–15]. Cognate ligands for PD-1 include PD-L1 and PD-L2. These are constitutively expressed on APCs and are induced in peripheral tissues during inflammatory responses or on the surface of tumor cells [13,16,17]. Among other inflammatory cytokines, interferon-gamma secreted during an immune response is a potent inducer of PD-L1 expression [18–20]. In contrast to CTLA-4, PD-1 is expressed on a broader range of immune cells (e.g., B lymphocytes and monocytes) [5,20–22]. And while PD-1 signaling is initiated during T cell activation, its primary effects of inducing CD8 + T cell anergy and, conversely, regulatory T cell activity and proliferation appear to be more pronounced during effector function in the peripheral tissues [5]. The critical roles of PD-1 and CTLA-4 in immune modulation were demonstrated in early studies that showed severe autoimmune pathologies in PD-1 and CTLA-4 knockout strains [22–24].

Although crucial in moderating inflammatory responses and preventing autoimmunity, checkpoint pathways can provide an immune evasion mechanism for tumors, allowing unchecked growth and progression. Preclinical studies have shown that PD-1 is expressed on a broad range of tumor infiltrating lymphocytes and is especially prominent on infiltrating T_regs_ and CD8 effector cells [25,26]. The PD-1 ligands, PDL1 and PDL2, are upregulated on a variety of tumor cells, and are also expressed by myeloid cells in the tumor microenvironment [27]. In studies by Dong et al., tumors expressing high levels of PD-L1 were found to promote apoptosis of tumor antigen-specific T cells in vitro as well as in mouse tumor models [17]. Early studies of antibody-mediated CTLA-4 blockade in a variety of transplantable tumor models (e.g., colon carcinoma, fibrosarcoma, ovarian cancer, and prostate cancer) demonstrated significant tumor response. An especially important finding in these studies was that once mice had experienced a response to CTLA-4 blockade, they were resistant to tumor rechallenge. These results demonstrated that, in addition to promoting regression of primary tumors, checkpoint inhibition facilitates the generation of an immunologic memory response that is associated with durable tumor remission [28,29].

These preclinical findings were validated in clinical trials of several immune checkpoint inhibitor antibodies [20]. In two large initial phase III trials, the anti-CTLA-4 monoclonal antibody ipilimumab significantly prolonged survival and produced durable responses in patients with advanced melanoma [1,30]. CTLA-4 blockade has also been shown to be active in patients with renal cell carcinoma and in patients with NSCLC [2,3]. Clinical studies of anti-PD-1 mAbs have also shown improvement in overall survival with durable responses in a variety of heavily pre-treated tumor types, including melanoma, NSCLC, and renal cell carcinoma [31]. Anti-PD1 mAbs have shown activity in hematologic malignancies as well, demonstrating a 66% ORR when combined with rituximab for follicular lymphoma [32], and a 51% ORR in patients with diffuse large B-cell lymphoma (DLBCL) who have progressed after autologous stem cell transplant [33]. Blockade of the PD-1 ligand PD-L1 has also shown activity in melanoma, renal cell cancer, and NSCLC, with overall response rates of 10–17% [34]. Importantly, a recent phase I trial demonstrated that the combination of PD-1 and CTLA-4 blockade produces greater than additive response rates in melanoma patients, with an ORR of 42% [35].

## 3 Adenosine-A2aR signaling: the emergence of a novel immune checkpoint pathway

While the clinical importance of immune checkpoints mediated by CTLA-4 and PD-1 has become clear, there are a number of other pathways active in the immune microenvironment that also appear to be important contributors to tumor immune evasion. While several of these pathways—like PD-1 and CTLA-4—are triggered by membrane-bound ligands (most notably LAG-3 and TIM-3 pathways), there are also soluble ligands found in the immune microenvironment that can function as triggers for checkpoint pathways [5]. Such soluble checkpoint ligands include tumor metabolites and cytokines such as IL-10 and TGF-beta [36]. Studies over the last two decades have also identified extracellular adenosine as a critical element in immune regulation [37–40].

### 3.1 Adenosine signaling through A2aR protects against exuberant immunologic response

Like CTLA-4 and PD-1, adenosine signaling in the inflammatory setting serves to dampen immunologic response and protect tissues from associated injury. While extracellular adenosine levels are typically very low, tissue breakdown and hypoxia (common to inflammatory and tumor microenvironments) generate high levels of extracellular adenosine [41,42]. Extracellular adenosine can signal through a set of four G-protein-coupled receptors: A1, A2a, A2b, and A3 [43]. Adenosine signaling through A2a and A2b receptors—expressed on a variety of immune cell subsets and endothelial cells—has been established as having an important role in protecting tissues during inflammatory responses [44–46]. Because of its distribution and dynamic expression pattern on a broader array of immune cells, most of this protective effect is thought to be secondary to signaling through the high-affinity A2a adenosine receptor. In a set of seminal experiments, Sitkovsky et al. showed that under physiologic conditions tissue injury is accompanied by A2aR-mediated accumulation of intracellular cAMP in immune cells [40]. These studies also noted a concomitant decrease in the release of pro-inflammatory cytokines (e.g., INF-gamma, TNF-alpha, IL-6). Genetic or pharmacologic blockade of the A2aR had profound effects on tissue inflammation, allowing for uncontrolled inflammatory response and tissue injury in mouse models of hepatitis and sepsis. A2aR-null mice experienced extensive tissue injury and death in inflammatory models that cause only minor, transient injury in wild type animals. Importantly, alternate inflammatory control mechanisms were unable to effectively compensate for the tissue damage resulting from the absence of A2aR signaling, thus establishing the adenosinergic pathway as a critical and non-redundant negative feedback control mechanism of inflammatory responses [40].

Subsequent experiments in our lab and others have confirmed the critical role of A2aR signaling in modulating tissue inflammation. In a mouse model of T cell mediated pneumonitis, A2aR signaling was found to significantly reduce tissue inflammation and prolong survival [7]. In these studies, a normally non-fatal pneumonitis caused by T cell transfer targeted to antigen-expressing lung tissue was found to be 80% fatal if the transferred T cells were from A2aR-null mice. Conversely, the effects of a normally lethal dose of A2aR-competent T-cells were almost completely abrogated by pharmacologic treatment with the A2aR-specific agonist CGS-21680. Thus, A2aR engagement provides an important tolerizing signal, which moderates tissue destruction and prolongs survival in the setting of T-cell mediated inflammation. Other studies have confirmed the non-redundant role for A2aR inflammatory modulation in a variety of other mouse models of inflammation, including sepsis, inflammatory bowel disease, and rheumatoid arthritis [38,47–49].

**Fig. 1:**
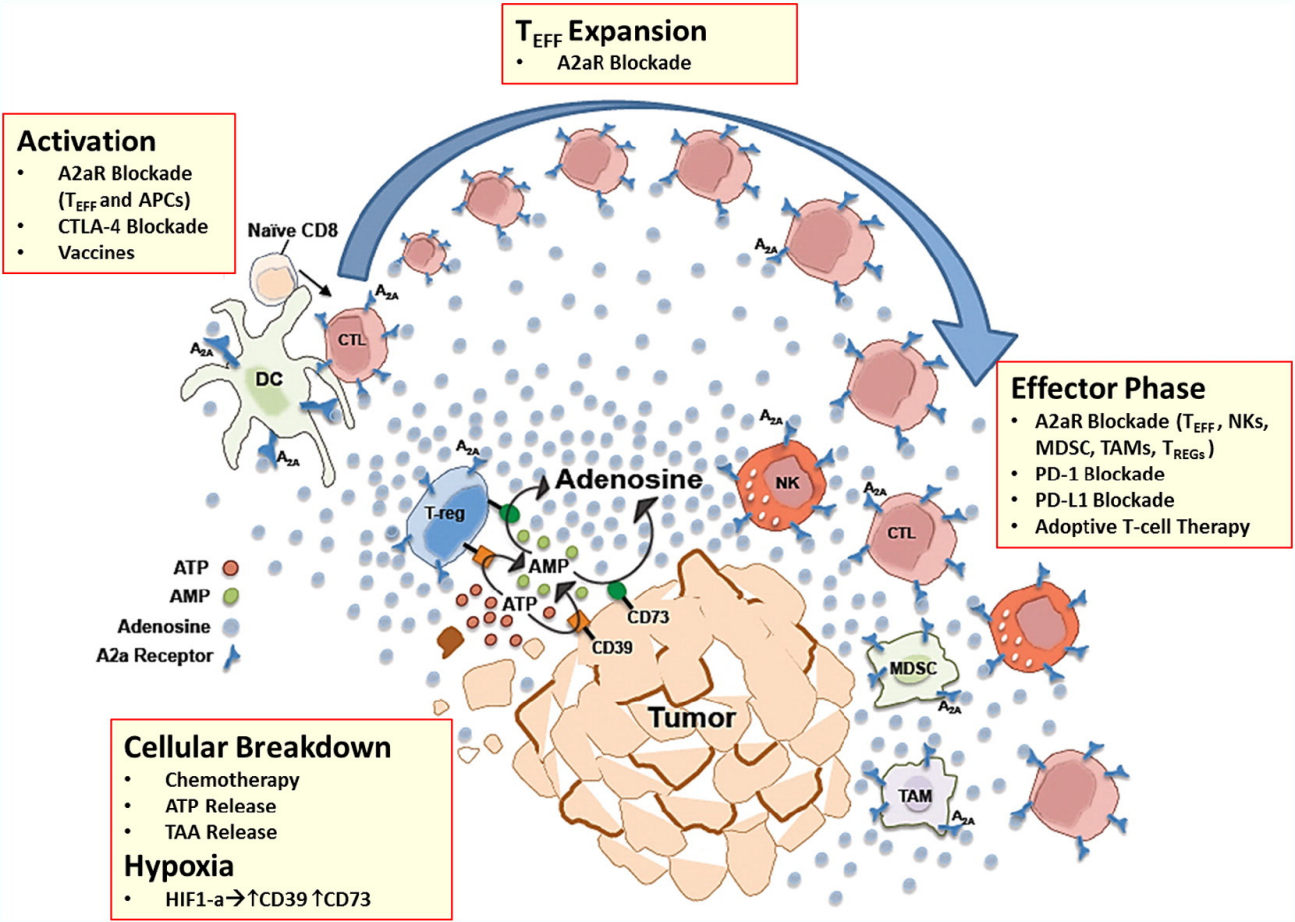
A2aR blockade in the tumor microenvironment. With increasing tumor cell breakdown in the setting of hypoxia, increased cellular stress, and chemotherapy, ATP, adenosine, and tumor associated antigens (TAA) are released into the tumor microenvironment (TME). ATP is further catabolized to adenosine by the ectonucleotidases CD39 and CD73, which are upregulated on a number of cell types within the TME, including regulatory T cells (T_regs_), stromal cells, and tumor cells. Adenosine in the TME has profound effects on all phases of immune function. Pharmacologic blockade of A2a receptors on effector T cells, T_regs_, NK cells, dendritic cells (DC), myeloid derived suppressor cells (MDSCs), and tumor-associated macrophages (TAMs) may counteract the immunosuppressive cloud of adenosine in the TME and enhance multiple phases of the immune response, including T cell activation, expansion, and effector function. Additive, and perhaps synergistic, effects may be possible by combining A2aR blockade with other modalities of cancer therapy. Chemotherapy, by causing increased cell turnover and breakdown, may allow exposure of hidden antigens and act as an in situ vaccine—an effect that may be enhanced by concomitant A2aR blockade to counteract associated elevations in extracellular adenosine levels. A2aR blockade has been shown to enhance the effect of tumor vaccines during T cell activation. A2aR inhibition may also work in concert with other immune checkpoint inhibitors, such as PD-1 or PD-L1 blockade, to further drive T cell function during the effector phase of immune response.

Through these and other studies a picture has emerged of adenosinergic signaling through A2aR as a negative feedback loop that regulates local and systemic inflammatory response. Under normal physiologic conditions extracellular release of adenosine is balanced by rapid cellular uptake that prevents a significant increase in extracellular levels [50,51]. In contrast, inflammatory environments and tumors produce high levels of extracellular ATP and adenosine [41,42,52]. As tissues are subjected to immune attack, increased cellular turnover and hypoxia trigger release of ATP and adenosine. While build-up of extracellular adenosine is partly a result of direct liberation of intracellular adenosine formed from increased ATP metabolism during cellular stress, levels are also increased by the catabolism of extracellular ATP and ADP by the tandem activity of the ectonucleotidases CD39 and CD73 (Fig. 1). In response to hypoxia-induced Hif1-alpha generation in tumors and inflamed tissues, CD39 and CD73 are upregulated on endothelial cells, stromal cells, some solid tumor cells and, importantly, on several subsets of immune cells, including T_regs_, CD8 + T cells, B cells, and others [6,46,53,54]. Elevated levels of extracellular adenosine activate specific purinergic receptors such as A2a (high affinity) and A2b (low affinity), which, as mentioned, have broad expression on immune cells and endothelial cells—the A2a receptor being a particular focus of attention given its higher affinity and wide distribution. A2a and A2b are G_s_ protein linked and trigger the accumulation of intracellular cAMP through stimulation of intracellular adenylyl cyclase [43,55,56]. The rise in intracellular cAMP—acting primarily through protein kinase A—has a broad range of immunosuppressive effects [57], including increased production of immunosuppressive cytokines (e.g., TGF-beta, IL-10) [7,58], upregulation of alternate immune checkpoint pathway receptors (e.g., PD-1, LAG-3) [7,59], increased FOXP3 expression in CD4 T cells driving a regulatory T cell phenotype, and induction of effector T cell anergy [7]. As in CTLA-4 and PD-1 pathways, significant influence of A2aR signaling on T_regs_ and effector T cells is likely the fundamental driving force of its immunosuppressive effect (though A2aR signaling on myeloid cells and NK cells likely also plays an important role). Since T_regs_ express high levels of CD39 and CD73, as CD4 + T cells are driven toward a T_reg_ phenotype by A2aR-mediated FOXP3 expression, an immunosuppressive amplification circuit generating increasing amounts of adenosine is created and quickly dampens the inflammatory response [60]. CD8 + effector cells, on the other hand, become less cytotoxic with decreased TCR signaling and increasingly anergic under the influence of A2aR signaling [7].

Given the importance of adenosinergic signaling in mediating negative feedback loops of immune responses, the effect of A2aR blockade on enhancing immunologic response has been investigated. In vivo studies in our lab utilizing A2aR knockout mice as well as studies using pharmacologic A2aR blockade, consistently demonstrate increased proliferative capacity and effector function of CD4 + and CD8 + T cells in response to activating antigen [61]. In fact, transient pharmacologic A2aR blockade in these studies was found to enhance immunologic memory, improving effector function several weeks after initial antigen challenge. Notably, this is not the case in A2aR-null mice, however, wherein persistent A2aR blockade eventually leads to an exhausted phenotype and disrupts transition to a memory phenotype (Waickman and Powell, unpublished findings). The difficulty in transitioning to a memory phenotype was also demonstrated in recent work by Cekic et al. [62]. In these studies, absence of A2aR signaling on A2aR-null lymphocytes hinders the accumulation of CD8 + effector-memory T cells in tumors in mouse models of melanoma and bladder cancer. In an earlier study by the same group, the absence of A2aR signaling was also shown to disrupt the homeostatic maintenance of the naïve T cell compartment, although it did not diminish the number of memory T cells in (non-tumor bearing) mice [63]. In this regard, A2aR signaling appears to attenuate the downregulation of the IL-7 receptor in response to TCR signaling through the PI3K-AKT pathway. Such signaling is important in both naïve T cell maintenance as well as transitioning to longer-lived phenotypes after initial T cell activation. It is important to note that these studies have examined the absence of A2aR signaling in knockout models and in the setting of irreversible A2aR blockade. As such, great care will be needed to optimize the dose and schedule of A2aR blockade within immunotherapeutic regimens.

### 3.2 A2aR blockade for immunotherapy in cancer

Analogous to CTLA-4 and PD-1, the immunologic dampening triggered by adenosine at sites of inflammation is mirrored by its effect in the tumor microenvironment. Several pioneering studies by Blay et al. allowed generalization of the idea of adenosine-mediated immunosuppression to the tumor microenvironment. In publications from the 1990s, this group theorized that supraphysiologic extracellular adenosine levels—driven by high cell turnover and hypoxia—could be responsible for observed immunosuppression in patients with solid tumors. In studies using a microdialysis probe it was demonstrated that extracellular adenosine levels in solid tumors were 10–20 times higher than adjacent tissues and reached levels sufficient to disrupt function of activated Cytotoxic T Lymphocytes (CTLs) [42]. During the same period, pioneering studies by Sitkovsky et al., began to uncover the critical interactions between extracellular ATP, adenosine and distinct subsets of immune cells [64–66]. Since that time, it has been found that, in addition to hypoxia and increased cell turnover, many cells in the tumor microenvironment (e.g., tumor cells, infiltrating immune cells, stromal cells, and endothelial cells) undergo ectopic expression of CD39 and CD73, further contributing to the buildup of extracellular adenosine [67,68]. In addition to dampening the effect of CTLs, increased extracellular adenosine has been found to down-modulate the activity of a range of immune functions in the tumor microenvironment, including the activity of macrophages, NK cells, neutrophils, and dendritic cells [69–73].

Given the similarities between adenosine-mediated immune modulation and established checkpoint pathways such as CTLA-4 and PD-1, the application of A2aR blockade to tumor immunotherapy is particularly exciting. In pioneering studies in 2006, Ohta et al. showed the complete rejection of two distinct tumor lines, CL8-1 melanoma and RMA T cell lymphoma, in a majority of A2aR null mice [4]. Notably, each of these tumor lines was 100% fatal in wild type mice. Responses in these models were dependent solely on CD8 + T cell activity. In another experiment, pharmacologic blockade of A2aR significantly augmented the tumor rejecting capacity of adoptively transferred, tumor-specific CD8 + T cells in a sarcoma model in mice [4]. They also showed the capacity for A2aR antagonism to strongly enhance CD8 + T cell-mediated destruction of the poorly immunogenic LL-LCMV tumor line. In studies by Beavis et al., A2aR antagonism was effective in reducing metastasis in CD73-expressing tumors in mouse models [74]. Included in these studies were investigations of the metastatic potential of the CD73-expressing murine breast cancer line, 4T1.2, as well as the melanoma line B16F10, which had been transduced to express CD73. Notably, in these studies NK cells were found to play a dominant role in limiting metastatic growth in these models.

Studies from our group have confirmed the increased capacity of A2aR^−/−^ mice to reject tumor cells in a variety of settings. In our initial tumor studies, A2aR^−/−^ mice showed significantly better tumor rejection and survival in a subcutaneous tumor model using the EL4 lymphoma cell line [61]. Interestingly, subcutaneous inoculation with a low-dose of EL4 lymphoma cells, which were readily rejected by both A2aR^−/−^ as well as wild type mice, allowed A2aR null mice to reject a subsequent challenge (on day 60) with an otherwise lethal dose of the same EL4 tumor line. Wild type mice in these experiments were unable to reject this re-challenge with tumor cells. This enhanced responsiveness was also elicited by vaccination with a 1:1 mixture of GMSF-secreting, irradiated melanoma cells (GVAX) and irradiated OVA peptide producing EL4 cells. In this case, the population of OVA-specific CD8 T cells in draining lymph nodes 7 days post inoculation was significantly elevated in A2aR null mice over wild type mice. In another experiment, GVAX inoculation was significantly more effective in protecting A2aR null mice from forming pulmonary lesions following subsequent (60 days after GVAX vaccine) tail vein injection of B16 melanoma cells.

An additional finding from our initial studies in A2aR null mice was the ability of A2aR blockade to synergize with inhibition of other checkpoint pathways [61]. Again using a subcutaneous EL4 model, A2aR-null mice exhibited longer tumor-free survival (TFS) and overall survival (OS) when treated with a soluble B7-DC/Fc fusion protein starting on the first day of tumor inoculation and continued for the length of the experiment. (B7-DC/Fc fusion protein acts as a ligand that specifically targets the PD-1 receptor expressed on dendritic cells and triggers profound T cell activation.) Improvement in TFS and OS were significant when compared to both untreated A2aR null mice as well as wild type mice with and without B7-DC/Fc. The increased effectiveness of A2aR blockade and concomitant PD-1 inhibition over either treatment alone was also seen in studies by Mittal et al., wherein metastases of CD73 + tumors was significantly decreased by combination therapy [75]. In these studies, Mittal et al. also demonstrated that A2aR blockade increases the activity of CTLA-4 and TIM-3 inhibition in controlling metastatic growth of CD73 + melanoma. Again, this group demonstrated a primary, though not exclusive, role for NK cells in metastatic control. In other studies of combination strategies, Iannone et al. showed that pharmacologic A2aR blockade can improve the efficacy of CTLA-4 therapy in mouse melanoma models. Of note, the efficacy of CTLA-4 inhibition in these studies was also enhanced by blockade of adenosine production upstream to A2aR by pharmacologic inhibition of CD73 activity [76].

As discussed above, CD73 and CD39 are ectonucleotidases that work in tandem to catabolize extracellular ATP to adenosine in the immune microenvironment. Though a complete review is beyond the scope of this article, investigations of CD73 blockade have shown significant effect on tumor control in mouse models and have also been especially effective in combination with both CTLA-4 inhibition and PD-1 blockade [53,77]. In studies of human tissue, CD73 expression on tumor cells was associated with chemotherapy resistance and poor overall prognosis in patients with triple-negative breast cancer [78]. A similar association has also been uncovered in several other types of cancer, including rectal carcinoma, gastric cancer, colorectal cancer, gallbladder cancer, chronic lymphoblastic leukemia, and prostate cancer [79]. These translational studies offer evidence of the importance of adenosine signaling in the tumor microenvironment in tumor progression. This idea has been bolstered by preclinical studies showing anti-CD73 mAb-induced reduction of primary tumors and metastases in two mouse models (4T1.2 and E0771) of breast cancer [80].

## 4 Translating A2aR blockade to tumor immunotherapy

With the clinical success of CTLA-4 and PD-1 checkpoint blockade in producing long-term responses in several distinct tumor types, there has been growing interest in understanding the specific determinants of host response during immunotherapy. As such, the critical parameters regarding immunologic response are being closely investigated, and it is becoming clear that future study of immunotherapeutic strategies will require assessment in a multitude of therapeutic and immunologic contexts. A single pathway, such as that triggered by extracellular adenosine, typically has multiple receptors, intra- and extracellular targets, and a range of distinct effects, all of which may depend on the specific developmental stage of a given target cell. As a case in point, a recent study found that A2a receptor blockade has distinct effects on T cell activation vs. effector-memory cell generation in a mouse melanoma model [62]. As mentioned, recent studies by Cekic, et al. have elucidated the importance of intact A2aR signaling for both maintenance of the naïve T cell compartment, as well as the transition to memory cell phenotypes in tumor-bearing mice. In these studies it was shown that *persistent* absence of A2aR signaling can actually stimulate tumor growth in some models [74,81]. Unpublished work from our lab confirms that, while transient blockade of A2aR signaling early in the immune response can drastically enhance the potency of a late recall response, complete elimination of A2aR signaling in knockout models appears to hinder efficient transition of CD4 + and CD8 + T cells to a memory phenotype. Further investigation of the importance of A2aR signaling in establishing, maintaining, or ameliorating anergy, exhaustion, and senescence of effector T cells will be informative avenues of inquiry.

Though there is certainly much work to be done in understanding the nuances of adenosinergic signaling on tumor immune response, the findings outlined in this review have a number of implications for clinical studies. Chief among these findings is the identification of adenosine-A2aR signaling as a critical and non-redundant negative regulator of inflammatory response that can be co-opted by tumors and function as a means of immune evasion. Signaling through this pathway has effects on activation, early expansion, and effector phases of T cell response. Furthermore, several preclinical studies have demonstrated the efficacy of A2a receptor inhibition in promoting tumor regression. In a number of studies A2aR blockade has been combined with other approaches to immunotherapy to potentiate additive effects on tumor control (Table 1).

As we move closer toward application of A2aR blockade in clinical trials, it is important to note that several A2a receptor antagonists have already gone through phase III trials for Parkinson Disease. These agents have generally been very well tolerated, without severe immune-related toxicities associated with CTLA-4 and PD-1 antagonism [82]. Recently reviewed by Pinna, these agents include Istradefylline, which has been approved for Parkinson Disease in Japan, as well as several agents presently in clinical trials (PBS-509, ST1535, ST4206, Tozadenant, V81444). Preladenant is an A2a receptor antagonist which has been discontinued after demonstrating poor efficacy in late phase clinical trials. Despite promising efficacy and a low incidence of adverse events, another A2aR antagonist, Vipadenant, was also discontinued after phase II studies [82].

### 4.1 Optimizing the immunotherapeutic effects of A2aR inhibition

While data from our lab and others show that A2aR blockade during initial T cell activation can greatly enhance T cell expansion and generation of memory phenotypes, studies by Ohta et al. show that A2aR blockade during adoptive T cell therapy in sarcoma models has a role in enhancing T cell effector function as well [4]. In addition, recent studies have shown that long-term A2aR blockade may interfere with the generation of immunologic memory [62]. Integrating these findings to achieve clinically effective A2aR inhibition will require careful consideration of the timing of blockade, as well as combination schemes using a range of other therapeutic approaches. In considering the importance of dosing, scheduling, and combination therapy, it is instructive to note that of the two initial CTLA-4 inhibitors, ipilimumab succeeded in phase III trials and garnered FDA approval whereas tremelimumab failed. This was despite the fact that these two agents showed equivalent intrinsic activity and phase II response rates [5]. The failure of tremelimumab in phase III studies is generally attributed to suboptimal dosing and scheduling, as well as other trial design flaws [1,5,83].

### 4.2 A2aR blockade during early immune response: combination therapy with vaccines and chemotherapy

Adenosine signaling has significant effects on several distinct cell types involved in the early stages of immune response. Specifically, while A2aR signaling on effector T cells decreases early post-activation proliferative capacity, A2aR signaling is also important in myeloid cells, polarizing professional APCs toward a more tolerogenic or suppressive phenotype and inhibiting the activation of effector cells (Fig. 1) [84–86]. The ability of A2aR blockade to reproducibly enhance vaccination strategies in a variety of tumor models confirms the robust effect of this pathway on T cell activation and early expansion. As many cancer vaccines have historically met with only limited success, adjunctive therapy with A2aR blockade may offer an important potentiating strategy (Table 2).

Largely through the work of Kroemer and Zitvogel, it has become increasingly clear that there is an immunologic component associated with the action of many cytotoxic chemotherapeutic agents [87–89]. In this regard, several chemotherapeutic agents, including anthracyclines, oxaliplatin, cyclophosphamide, gemcitabine, and bortezomib appear to produce an in situ vaccination as a consequence of their initial cytotoxic effect. In so doing, these agents appear to facilitate an immunogenic cell death, which has several important attributes [87–91]. Immunogenic cell death, as defined by Kroemer et al., is a process that stimulates an immune response against dead cell antigens (tumor antigens) through the timed release of soluble mediators as well as early changes on the surface of cancer cells. Interestingly, the release of ATP has been identified as a critical mediator in this process. While ATP acts as immunostimulant, facilitating the recruitment of dendritic cells into the tumor bed, it is eventually catabolized to adenosine by ectonucleotidases CD39 and CD73 that are often highly expressed in the tumor microenvironment. As such, the immunostimulatory effects of ATP give way to the immunosuppressive effects of adenosine. This presents an excellent opportunity for concomitant A2aR blockade. A2aR antagonism during chemotherapy may allow the expansion of tumor-specific T cells, and simultaneously repress the induction of tumor-specific regulatory T cells, thus helping to kindle the immunologic response. To this end, the work of Zitvogel and Kroemer, as well as the work by Stagg and others, have shown the effectiveness of combining adenosinergic signaling blockade in the context of cytotoxic chemotherapy [90,91]. Stagg et al. demonstrated the success of this approach by inhibiting adenosine production (upstream of A2aR) with CD73 blockade in combination with doxorubicin chemotherapy in a murine breast cancer model [78]. CD73 blockade in these experiments enhanced antitumor immune response, especially when given in combination with doxorubicin, prolonging survival in mice with established metastatic breast cancer compared with either agent given as monotherapy. In this work, a similar effect was also observed when a specific A2aR blocking agent, SCH58261, is used in combination with doxorubicin. Similar studies examining pharmacologic blockade of A2aR in combination with chemotherapy are ongoing in our lab.

### 4.3 A2aR blockade in the context of multiple checkpoint pathway inhibition

The ability of A2aR pathway blockade to produce additive effects in combination with targeting of other checkpoint pathways has mechanistic as well as clinical implications. Mechanistically, studies showing an additive response underline the independence of the adenosinergic-A2aR pathway from established checkpoint pathways. Clinically, the non-redundant nature of these pathways implies that combination checkpoint pathway inhibition, including adenosinergic blockade, can have potentially dramatic effects on response rates. To that end, recent trials combining CTLA-4 and PD-1 blockade reported initial findings of an overall response rate of 42%—significantly higher than either agent used alone [35]. While CTLA-4 blockade appears to be most effective in enhancing the activation phase of cellular immune response, whereas PD-1 inhibition is most profound during the effector phase [5], the addition of A2aR blockade has the potential to further lower the threshold for each of these critical immune events (Table 1). In this regard, it is possible that concomitant use of A2a receptor antagonism with CTLA-4 or PD-1 may allow for dose reductions of either agent, thereby reducing the incidence and severity of immune related toxicities.

### 4.4 A2aR blockade during effector phase of the immune response: combination therapy with adoptive T cell therapy

The ability of A2aR blockade to enhance effector function is an important aspect of its mode of action. Adenosine signaling through A2aR has suppressive effects on both CD4 + and CD8 + effector T cell compartments, including: polarization of CD4 + cells away from the Th1 phenotype; decreased production of IFN-gamma, IL-2, and TNF-alpha; reduced cytoxicity of CTLs; reduced TCR signaling; and reduced CTL activity leading to increased anergy [4,7,57,59,92]. This has been confirmed in preclinical studies in which A2aR inhibition has demonstrated the ability to enhance effector function during an immune response (Fig. 1) [4,7,40]. Given these properties, we expect that the combination of A2aR blockade with adoptive T cell therapy will generate enhanced T cell function and extended duration of cytotoxic response (Table 2). As mentioned, early studies by Ohta et al. specifically demonstrated the benefit of A2aR blockade in mouse tumor models using adoptive T cell therapy [4].

### 4.5 A2aR blockade in combination with other targets in the adenosinergic pathway

Lastly, while the A2a adenosine receptor is an attractive target for tumor immunotherapy, inhibition of other targets in the adenosinergic pathway has also yielded encouraging results. Of particular interest has been the upstream ectonucleotidase CD73. Several groups have shown that CD73 blockade can have dramatic effects on both primary tumor response as well as metastatic processes [93–95]. As mentioned, the level of CD73 in triple-negative breast cancer tissues was found to be negatively correlated with prognosis and response to chemotherapy [78]. Recently, CD73 expression in tumor tissue has also been correlated with poor prognosis in rectal adenocarcinoma [96]. Several other studies have also found an association between CD73 expression in tumor tissue and more aggressive clinical behavior, including studies in gastric cancer, colorectal cancer, gallbladder cancer, chronic lymphoblastic leukemia, and prostate cancer [79]. Studies in mouse models using CD73-null mice have shown increased tumor immunity in a variety of tumor types, including MC38 colon cancer, EG7 lymphoma, AT-3 mammary tumors, ID8 ovarian tumors, and B16F10 melanoma [77,94,97]. CD73 blockade with both small molecules and anti-CD73 mAb has shown specific responses in mouse models of B16 melanoma and 4T1.2 breast cancer [77,97]. Also, inhibition of another adenosine receptor, A2bR, has been shown to inhibit growth of prostate cancer cell lines (though not through an immunologic mechanism) [98]. In this regard, Stagg et al.showed that A2bR activation promoted metastatic cancer cell phenotype in a 4T1.2 mouse model of breast cancer [80]. Furthermore, Cekic et al. demonstrated enhanced activation of dendritic cells and improved CXCR3-dependent T cell tumor infiltration in the setting of pharmacologic A2bR blockade [99]. In these studies, specific A2bR blockade with ATL801 slowed tumor growth in mouse models of bladder and breast cancer. It remains to be seen if simultaneous inhibition of several members of the adenosinergic pathway can produce non-redundant effects on tumor response.

## 5 Conclusion

With the recent clinical success in applying CTLA-4 and PD-1 blockade to the treatment of a variety of tumors, the promise of cancer immunotherapy has begun to be realized. In targeting the maladaptive appropriation of immune checkpoints in the tumor microenvironment and not the cancer directly, these treatments may represent a sea change in the approach to treatment of many cancers. In addition to providing significant response rates in patients with highly pretreated and refractory tumors, the establishment of immunologic memory has generated durable responses in many of these patients. Given the impressive results of CTLA-4 and PD-1 inhibition in cancer patients, other checkpoint pathways operating within the tumor environment demand thorough investigation. Clearly, a challenge for the future will be to determine the most effective integration of A2aR inhibitors in terms of dosing and timing within various combination regimens. Additionally, while this review has mostly focused on the role of A2aR signaling on T cells, it is clear that A2aR blockade will also promote tumor immunotherapy through its effect of NK cells, as well as myeloid derived suppressor cells, and tumor-associated macrophages.

## Figures and Tables

**Table 1:** A2aR blockade in murine models of cancer.

A2aR inhibitor	Effect observed
SCH58261	Enhanced tumor immunotherapy in combination with PD-1 inhibition in CD73 expressing tumors [74] Suppression of metastases in CD73 + tumor models [81] Prolonged survival and reduction of metastatic burden in melanoma and breast cancer mouse models in combination with anti-PD1 mAb [75] Enhanced doxorubicin sensitivity in CD73 expressing 4T1.2 breast cancer tumors resulting in improved tumor control [78]
SYN115	Enhanced tumor immunotherapy in combination with anti-PD-1 mAb in CD73 expressing tumors [81]
ZM241365	In combination with anti-CTLA4 mAb inhibited tumor growth and enhanced anti-tumor immune responses in B16F10 mouse melanoma model [76]
FSPTP (irreversible inhibitor)	Intratumoral injection reduced frequency of tumor infiltrating CD8 + T cells, but not CD4 + T cells or NK cells, in MB49 bladder cancer model [62]

**Table 2:** Potential therapeutic applications of A2a receptor blockade.

1. Tumor vaccines + A2aR blockade	A2aR blockade during the peri-vaccination period to enhance activation and subsequent expansion of activated effector cells
2. Chemotherapy + A2aR blockade	A2aR blockade during chemotherapy to enhance in situ vaccination and counteract elevated extracellular adenosine levels resulting for increased cell turnover
3. PD-1/PD-L1/CTLA-4 + A2aR blockade	A2aR blockade in combination with established immune checkpoint inhibition to enhance activation and effector function of cellular immune components
4. Adoptive T cell therapy + A2aR blockade	A2aR blockade during the effector phase to enhance T cell function and extend the duration of cytotoxic response
